# Antigenic Characterization of Novel Human Norovirus GII.4 Variants San Francisco 2017 and Hong Kong 2019

**DOI:** 10.3201/eid3005.231694

**Published:** 2024-05

**Authors:** Kentaro Tohma, Michael Landivar, Lauren A. Ford-Siltz, Kelsey A. Pilewski, Joseph A. Kendra, Sandra Niendorf, Gabriel I. Parra

**Affiliations:** US Food and Drug Administration Center for Biologics Evaluation and Research, Silver Spring, Maryland, USA (K. Tohma, M. Landivar, L.A. Ford-Siltz, K.A. Pilewski, J.A. Kendra, G.I. Parra);; Robert Koch Institute, Berlin, Germany (S. Niendorf)

**Keywords:** viruses, norovirus, acute gastroenteritis, GII.4 variants, Hong Kong 2019 variant, San Francisco 2017 variant, enteric infections, antigenicity

## Abstract

Norovirus is a major cause of acute gastroenteritis; GII.4 is the predominant strain in humans. Recently, 2 new GII.4 variants, Hong Kong 2019 and San Francisco 2017, were reported. Characterization using GII.4 monoclonal antibodies and serum demonstrated different antigenic profiles for the new variants compared with historical variants.

Norovirus is a major cause of acute gastroenteritis (*1*). Over past decades, variants of the predominant genotype, genotype 4 (GII.4), have continuously emerged to escape immunity (*2*–*5*). Since 2012, the Sydney 2012 variant has predominated worldwide (*6*). In 2019, GII.4 noroviruses that did not cluster with any known variants were reported circulating in different countries as early as 2016 (*7*,*8*). Based on phylogenetic clustering and number of mutations on major capsid viral proteins (VP1), the variant was classified GII.4 Hong Kong 2019 (*7*). The new variant caused no large outbreaks and did not eclipse the predominance of Sydney 2012. Another recently reported unique group of GII.4 noroviruses, the San Francisco 2017 variant, was retrospectively detected circulating during 2017–2022 (*9*). Both variants showed multiple mutations on major antigenic sites, including a single amino acid insertion next to the antigenic site A in San Francisco 2017 (*7*,*9*,*10*). We characterize the antigenicity of these 2 new variants using panels of GII.4 mouse monoclonal antibodies (mAbs) and hyperimmune serum developed against historical GII.4 variants (*11*,*12*).

## The Study

**Figure 1:**
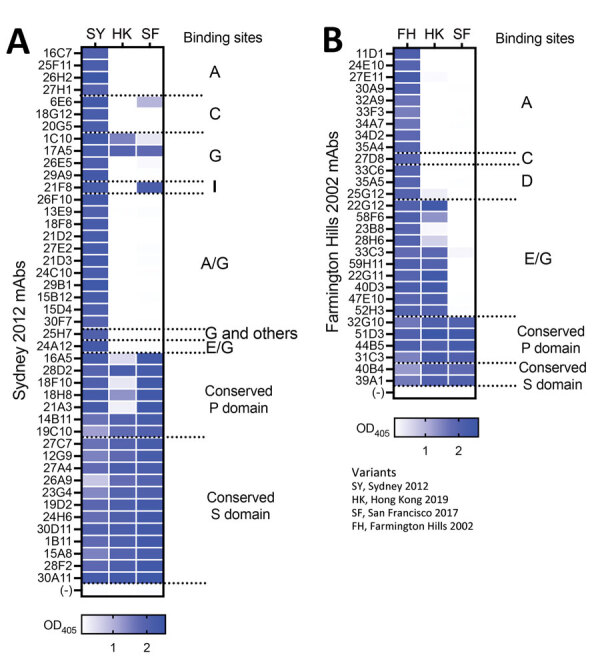
Monoclonal antibodies raised against 2 major GII.4 variants in a study of novel human norovirus GII.4 variants, San Francisco 2017 and Hong Kong 2019. A) Sydney 2012 mAb panel; B) Farmington Hills 2002 mAb panel. The heatmaps indicate ELISA binding strength (OD_405_ values) of individual mAbs against virus-like particles from GII.4 Hong Kong 2019 and San Francisco 2017. Antibodies indicate minimal cross-reactivity between new and previously described variants. The binding sites of the mAbs were characterized in a previous study (*11*). mAbs, monoclonal antibodies; OD_405_, optical density at 405 nm; P, protruding; S, shell.

To determine the cross-reactivity of the 2 new variants with previously circulating variants, we produced virus-like particles (VLPs) for Hong Kong 2019 (GenBank accession no.: MN400355) and San Francisco 2017 (GenBank accession no. MW506849) viruses. We performed ELISA by using mAbs developed against Sydney 2012 virus and the newly developed VLPs (*11*). Results demonstrated that the Hong Kong 2019 VLPs bound to most of the mAbs mapping to conserved sites from protruding (P) and shell (S) domains of the VP1, but only bound to 2/25 mAbs that mapped to variable antigenic sites and showed histo-blood group antigen (HBGA) blockade activity (Figure 1, panel A) (*11*). Those results were expected because mutational analyses showed that the Hong Kong 2019 viruses present multiple mutations on variable antigenic sites (Appendix) (*7*). The loss of binding of 3 cross-reactive mAbs that mapped to the P domain could be explained by unique mutations on the conserved sites (Appendix). VLPs from the San Francisco 2017 variant bound to all mAbs mapping to conserved sites of the VP1, but only to 4 mAbs that mapped to variable antigenic sites. Based on previous observations, alanine on positions 356, 359, or both, play a role in binding to mAbs 1C10 and 17A5 (*11*). Thus, alanine on those positions could explain the binding of mAbs to Hong Kong 2019 and San Francisco 2017 VLPs. Other mAbs mapping to the antigenic site G, 26E5 and 29A9, seem to require residues from antigenic site A (*11*). Mutations on antigenic site A in San Francisco 2017 could therefore result in loss of binding of these mAbs regardless of similarity to antigenic site G on the Sydney 2012 variant. The 6E6 mAb, mapping to antigenic site C, was previously reported to cross-react weakly to Farmington Hills 2002 variant (*11*), which has 3 mutations compared with Sydney 2012. The San Francisco 2017 presented 4 mutations compared with Sydney 2012; the Hong Kong 2019 variant had 6 mutations on that site, explaining the differential binding of this mAb. Similarly, sequence differences on antigenic site I could explain the lack of binding of mAb 21F8 to Hong Kong 2019 VLPs. 

Because Hong Kong 2019 and San Francisco 2017 present evolutionary convergence and share similar residues on several of the antigenic sites compared with the Farmington Hills 2002 variant (Appendix), we also tested those strains with mAbs developed against this ancestral variant (Figure 1, panel B). Both VLPs showed reactivity with all mAbs binding to conserved epitopes. As expected based on sequence similarity, Hong Kong 2019 showed reactivity only with mAbs mapping on antigenic site E/G. San Francisco 2017 was negative to all mAbs mapping to variable sites, including antigenic site A, which presented only 3 mutations from Farmington Hills 2002 VLPs. Those data indicate that either a small number of changes are sufficient to abrogate binding of all 9 A-mapping mAbs or that the insertion near the antigenic site A has a major influence on the characteristics of this antigenic site. 

**Figure 2:**
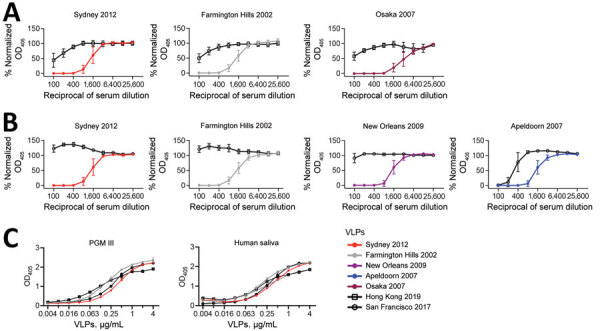
HBGA blockade and binding assays in a study of novel human norovirus GII.4 variants, San Francisco 2017 and Hong Kong 2019. A,B) Line graphs indicating normalized OD_405_ curves of GII.4 variants in HBGA blockade assays using mouse hyperimmune serum raised against currently circulating strains; A) Hong Kong 2019 VLPs against historical strains; B) San Francisco 2017 VLPs against historical strains. Normalized OD_405_ values were calculated by using values from positive and negative (serum only) control wells. C) OD_405_ curves of GII.4 variant VLPs in HGBA binding assays of Hong Kong 2019 and San Francisco 2017 VLPs and PGM III and human saliva, expressing the Lewis^a^, Lewis^b^, Lewis^y^, H type-1, and H type-2 HBGA carbohydrates. PGM III was used as a source of HBGA carbohydrates. Human saliva was collected from a healthy adult volunteer under US Food and Drug Administration, Center for Biologics Evaluation and Research protocol no. CBER IRB 16–069B. HBGA, histo-blood group antigen; OD_405_, optical density at 405 nm; PGM, porcine gastric mucin; VLP, virus-like particles.

To further characterize the antigenicity of these new variants, we tested the HBGA blocking activity of serum from mice immunized with VLPs from historical variants (Figure 2, panels A, B) (*12*), including the currently circulating Sydney 2012, the ancestral Farmington Hills 2002, and genetically or phylogenetically related variants: Osaka 2007 for Hong Kong 2019 viruses (*7*), and New Orleans 2009 and Apeldoorn 2007 for San Francisco 2017 viruses (*9*) (Appendix). The Hong Kong 2019 VLPs presented weak cross-blockade reactivity with the serum raised against all 3 viruses (mean 50% effective concentration = 118.5 for Sydney 2012, 116.9 for Farmington Hills 2002, and 87.6 for Osaka 2007 serum), with >12-fold differences compared with their homologous VLPs (Figure 2, panel A). That result was consistent with data from children with the fewest previous norovirus infections (*10*), supporting minimal cross-reactivity of Hong Kong 2019 to previous variants. In contrast, the San Francisco 2017 VLPs did not show cross-blockade reactivity with any of the serum samples tested from pandemic variants (Figure 2, panel B), including Sydney 2012 and Farmington Hills 2002, that shared similar sequences on the antigenic sites E/G (Sydney 2012) and A (Farmington Hills 2002) (Appendix). The only exception for cross-reactivity was with the serum raised against Apeldoorn 2007, which showed moderate cross-blockade activity with the San Francisco 2017 (50% effective concentration = 309.2, a 5-fold difference compared with homologous VLPs). Of note, GII.4 Apeldoorn 2007 and San Francisco 2017 share the same motif on the antigenic site D (Appendix). Thus, that cross-reactivity might be explained by antibodies mapping on the antigenic site D from Apeldoorn 2007 variant. 

Our data indicate that both Hong Kong 2019 and San Francisco 2017 variants present distinct antigenic profiles, yet both viruses have been circulating for >7 years without causing large outbreaks globally. Multiple examples of antigenically distinct noroviruses that spread worldwide without causing large outbreaks exist. Minor variants such as Osaka 2007 and Apeldoorn 2007 showed distinct antigenic profiles to variants that circulated before their emergence (*12*). Those variants caused local outbreaks and spread to multiple countries, but none predominated at the global level (*13*). Therefore, changes in antigenicity might not be the only factor determining the epidemic potential of noroviruses. Indeed, specific HBGA binding profiles were associated with emerging noroviruses (*14*,*15*). The new GII.4 variants bound to porcine gastric mucin III and human saliva, as did other current and archival variants (*9*,*10*) (Figure 2, panel C), suggesting that impairment of binding to HBGA did not cause the lower circulation of these viruses. 

In conclusion, these 2 new norovirus variants are antigenically distinct from previously circulating variants. Whether these variants will predominate or are examples of the subdued circulation of minor norovirus variants remains to be determined. To prepare for future pandemics, we must delineate the factors that determine the overall fitness and predominance of GII.4 noroviruses, including but not limited to replication kinetics, pathogenicity, HBGA binding spectrum, and epidemiologic confounder.

Appendix Additional information about a study of novel human norovirus GII.4 variants, San Francisco 2017 and Hong Kong 2019.
