# Supplementary material for: Antigenic Characterization of Novel Human Norovirus GII.4 Variants San Francisco 2017 and Hong Kong 2019
Source: Emerg Infect Dis. 2024 May;30(5):1026–9. doi: 10.3201/eid3005.231694 (PMC11060466; doi:10.3201/eid3005.231694)
Supplement: Appendix — Additional information about a study of novel human norovirus GII.4 variants, San Francisco 2017 and Hong Kong 2019. [file 23-1694-Techapp-s1.pdf]

Article DOI: https://doi.org/10.3201/eid3005.231694

EID cannot ensure accessibility for Appendix materials supplied by authors. Readers who have difficulty accessing Appendix content should contact the authors for assistance.

Antigenic Characterization of Novel Human Norovirus GII.4 Variants, San Francisco 2017 and Hong Kong 2019

Appendix

Genetic Analyses

The emergence of GII.4 variants was associated with multiple mutations mapping to five major antigenic sites (namely, A, C, D, E, and G) on the major capsid protein, VP1 (*1*,*2*). These antigenic sites are involved in viral neutralization and blockade of interactions between the VP1 and histo-blood group antigen (HBGA) carbohydrates (*3*), which are ligands that facilitate norovirus infection (*4*–*7*). This Appendix provides a comprehensive mutational analysis of new GII.4 variants using a large, n = 3,142, dataset of complete GII.4 VP1 sequences (*8*), and the viruses classified as GII.4 Hong Kong 2019 (*9*) and GII.4 San Francisco 2017 (*10*). The phylogenetic relationship of new and historical GII.4 variants were estimated using maximum-likelihood method using VP1 amino acid sequences. In the phylogenetic analysis, the dataset was downsized to consist of randomly subsampled sequences with a maximum of 30 sequences per variant. The phylogenetic tree was calculated using the best-fit substitution model as implemented in IQ-TREE (*11*). The number of nucleotide or amino acid changes between previous GII.4 variants and new variants was measured using *phylotools* and *utils* packages and consensus sequence from each variant was estimated using *seqinr* package in R (*12*).

As indicated in previous studies (*9*,*10*), the phylogenetic tree showed that Hong Kong 2019 and San Francisco 2017 variants are distinct from other GII.4 variants (Appendix Figure 1). The Hong Kong 2019 is most closely related to the Osaka 2007 variant, while San Francisco 2017 branched out from Sydney 2012 variants. The mutational analysis of the new variants confirmed multiple substitutions on the major antigenic sites A, C, D, E, and G as indicated in previous studies (*9*,*10*,*13*), and on other subdominant sites, namely I, resulting in different amino

acid sequence patterns (Appendix Figures 2, 3). Hong Kong 2019 showed unique patterns on antigenic sites A, D, I, and multiple other positions on the P domain. As expected by the phylogenetic analysis (*9*) (Appendix Figure 1), this virus showed sequence similarities with Osaka 2007 on the major antigenic sites. Notably, Hong Kong 2019 displayed the same amino acid sequence on antigenic site E from the ancestral Farmington Hills 2002, but the overall difference between these two viruses is marked by ≥4 amino acid changes on each of the other four (A, C, D, G) antigenic sites (Appendix Figure 2).

In addition to an insertion described between positions 293 and 294 (*10*), the San Francisco 2017 variant contained multiple amino acid mutations on antigenic site A as compared with Sydney 2012 (median: 3 mutations out of 8 residues) and Apeldoorn 2007 (median: 4 mutations), but ≤2 mutations on antigenic sites D, E, and G (Appendix Figure 2). Interestingly, San Francisco 2017 presented large intra-variant variations on antigenic site A, and 6 out of 15 San Francisco 2017 strains reported in previous study (*10*) shared same sequence on this antigenic site with Sydney 2012 viruses that were detected in late 2010s and 2020s (*8*). These late Sydney 2012 viruses showed evolutionary convergence into the ancestral GII.4 variants on antigenic site A (*8*) (Appendix Figure 4). Likewise, San Francisco 2017 presented a similar antigenic site A (median: 3 mutations) to ancestral Grimsby 1995 or Farmington Hills 2002 variants, but a completely different antigenic site G (median: ≥5 mutations out of 6 residues) (Appendix Figure 2). Although these variants shared similarity on antigenic site A, their consensus nucleotide sequences indicated a large divergence between them. Thus, the San Francisco 2017 consensus sequence displayed 5–7 nucleotide but only 2 amino acid changes as compared to the Farmington Hills 2002 and Grimsby 1995 consensus sequences of antigenic site A (Appendix Figure 4). When comparing the nucleotide sequence of these codon positions with phylogenetically closer variants (Apeldoorn 2007, early and late Sydney 2012 viruses), the San Francisco 2017 viruses presented 4–6 nucleotide changes that resulted in 2–4 amino acid mutations. Of note, consensus San Francisco 2017 presented a different codon pattern on position 368 (GAA) as compared with Farmington Hills 2002 (AAC) and Grimsby 1995 (ACC), which is same codon (GAA) with early and late Sydney 2012 viruses. Together, these data suggest that the San Francisco 2017 variant converged into similar amino acids on the antigenic site A present in the ancestral variants via different evolutionary pathways, confirming previous

observations that there are constraints on the use of residues throughout the evolution of GII.4 norovirus (*8*).

In conclusion, the new variants presented multiple amino acid changes on the major antigenic sites when compared with all previously described viruses, with several of them converging into motifs observed in previous variants. A description of effect of these mutations on the antigenic profile of these variants is presented in the main text.

References

1. Tohma K, Lepore CJ, Gao Y, Ford-Siltz LA, Parra GI. Population genomics of GII.4 noroviruses reveal complex diversification and new antigenic sites involved in the emergence of pandemic strains. mBio. 2019;10:10. PubMed https://doi.org/10.1128/mBio.02202-19

2. Lindesmith LC, Costantini V, Swanstrom J, Debbink K, Donaldson EF, Vinje J, et al. Emergence of a norovirus GII.4 strain correlates with changes in evolving blockade epitopes. J Virol. 2013;87:2803–13. PubMed https://doi.org/10.1128/JVI.03106-12

3. Tohma K, Ford-Siltz LA, Kendra JA, Parra GI. Dynamic immunodominance hierarchy of neutralizing antibody responses to evolving GII.4 noroviruses. Cell Rep. 2022;39:110689. PubMed https://doi.org/10.1016/j.celrep.2022.110689

4. Haga K, Ettayebi K, Tenge VR, Karandikar UC, Lewis MA, Lin SC, et al. Genetic manipulation of human intestinal enteroids demonstrates the necessity of a functional fucosyltransferase 2 gene for secretor-dependent human norovirus infection. mBio. 2020;11:e00251-20.

5. Marionneau S, Ruvoen N, Le Moullac-Vaidye B, Clement M, Cailleau-Thomas A, Ruiz-Palacois G, et al. Norwalk virus binds to histo-blood group antigens present on gastroduodenal epithelial cells of secretor individuals. Gastroenterology. 2002;122:1967–77.

6. Tan M, Huang P, Meller J, Zhong W, Farkas T, Jiang X. Mutations within the P2 domain of norovirus capsid affect binding to human histo-blood group antigens: evidence for a binding pocket. J Virol. 2003;77:12562–71. PubMed https://doi.org/10.1128/JVI.77.23.12562-12571.2003

7. Rockx BHG, Vennema H, Hoebe CJPA, Duizer E, Koopmans MPG. Association of histo-blood group antigens and susceptibility to norovirus infections. J Infect Dis. 2005;191:749–54. https://doi.org/10.1086/427779

8. Parra GI, Tohma K, Ford-Siltz LA, Eguino P, Kendra JA, Pilewski KA, et al. Minimal antigenic evolution after a decade of norovirus GII.4 Sydney_2012 circulation in humans. J Virol. 2023;97:e0171622. PubMed https://doi.org/10.1128/jvi.01716-22

9. Chan MC, Roy S, Bonifacio J, Zhang LY, Chhabra P, Chan JCM, et al.; for NOROPATROL2. Detection of norovirus variant GII.4 Hong Kong in Asia and Europe, 2017–2019. Emerg Infect Dis. 2021;27:289–93. PubMed https://doi.org/10.3201/eid2701.203351

10. Chhabra P, Tully DC, Mans J, Niendorf S, Barclay L, Cannon JL, et al. Emergence of novel norovirus GII.4 variant. Emerg Infect Dis. 2024;30:163–7. PubMed https://doi.org/10.3201/eid3001.231003

11. Trifinopoulos J, Nguyen LT, von Haeseler A, Minh BQ. W-IQ-TREE: a fast online phylogenetic tool for maximum likelihood analysis. Nucleic Acids Res. 2016;44(W1):W232–5.

12. R Core Team. R: a language and environment for statistical computing. Vienna, Austria: R Foundation for Statistical Computing; 2023.

13. Lindesmith LC, Boshier FAT, Brewer-Jensen PD, Roy S, Costantini V, Mallory ML, et al. Immune imprinting drives human norovirus potential for global spread. mBio. 2022;13:e0186122. PubMed https://doi.org/10.1128/mbio.01861-22

14. Kendra JA, Tohma K, Ford-Siltz LA, Lepore CJ, Parra GI. Antigenic cartography reveals complexities of genetic determinants that lead to antigenic differences among pandemic GII.4 noroviruses. Proc Natl Acad Sci U S A. 2021;118:e2015874118. PubMed https://doi.org/10.1073/pnas.2015874118

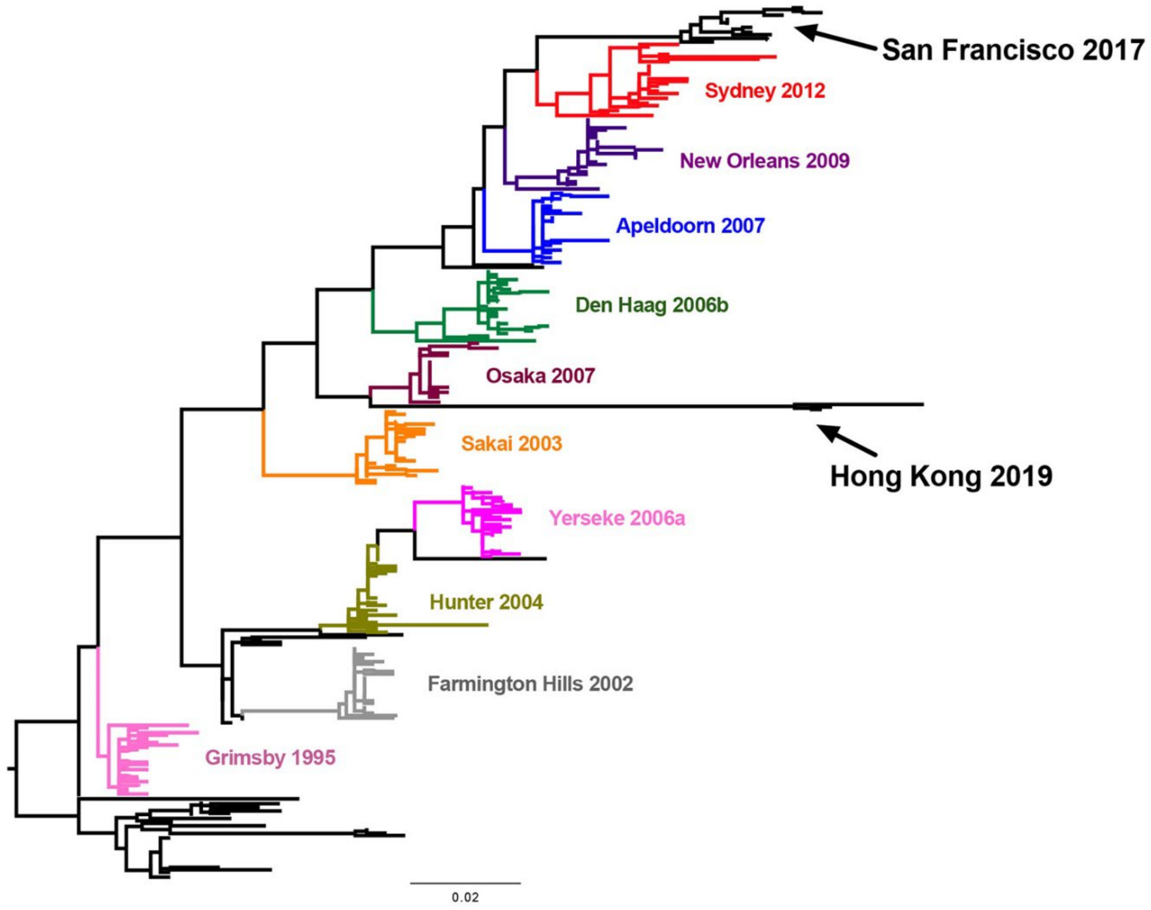

Appendix Figure 1. Phylogenetic tree showing the evolutionary relationship of the new variants, GII.4 Hong Kong 2019 and San Francisco 2017, with previously described GII.4 variants. The phylogenetic tree was calculated with the maximum-likelihood method and randomly sampled (n = 338) VP1 sequences with sequences from new variants, Hong Kong 2019 and San Francisco 2017. Scale bar indicates the genetic distance, measured by the number of amino acid substitutions per site.

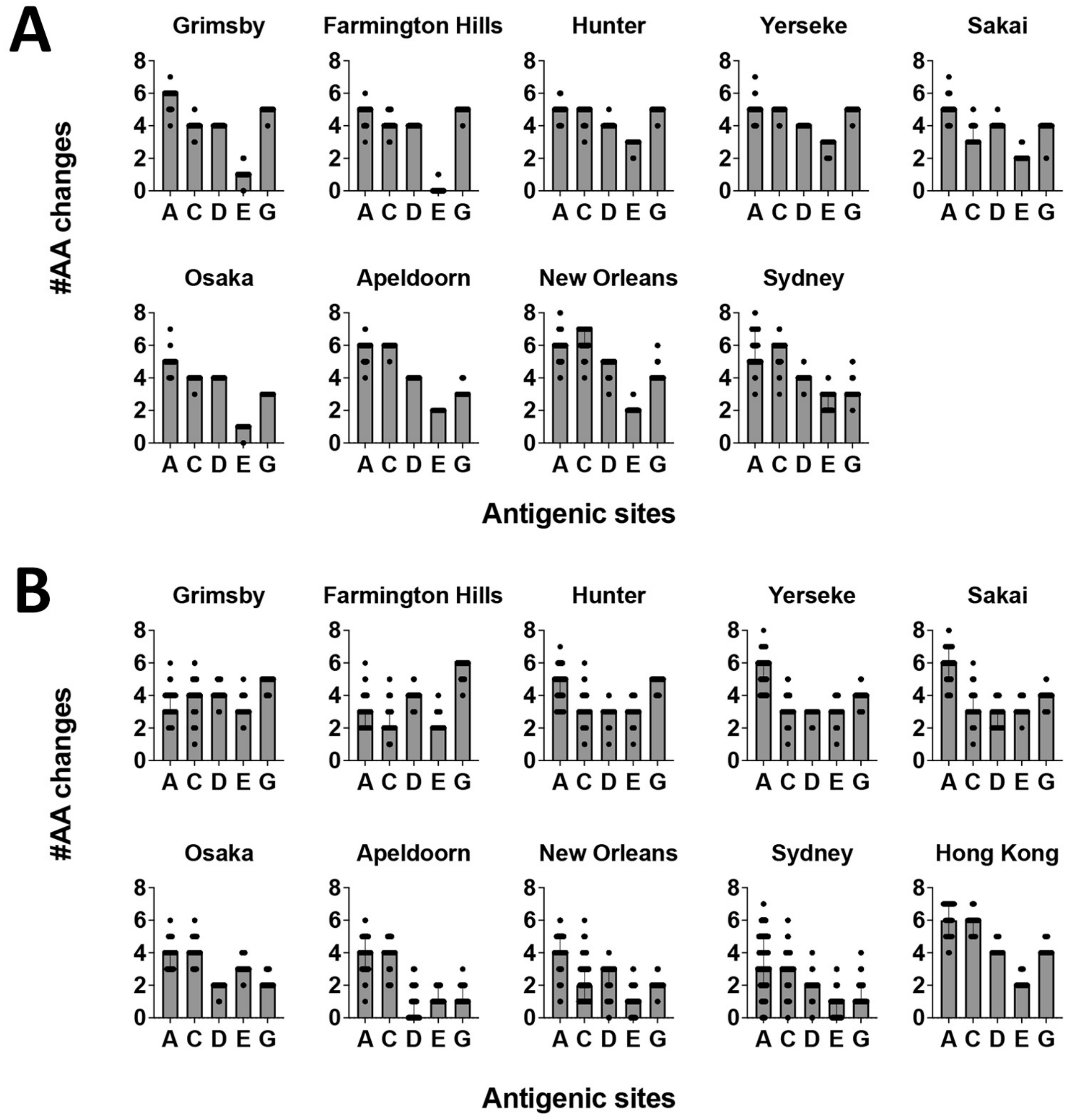

Appendix Figure 2. Number of amino acid mutations on individual major antigenic sites between new and previous variants. The bar graphs show the number of amino acid mutations on each antigenic site between Hong Kong 2019 (A) or San Francisco 2017 (B) and historical GII.4 variants (n = 3,142). The bar and error bars indicate median and interquartile range. AA, amino acid.

Variant	Strain (accession number)	pre-A			Major antigenic sites involved in HBGA-blockade and neutralization														
					A								C						
		292	293	Insertion	294	295	296	297	298	368	372	373	339	340	341	375	376	377	378
Hong Kong	CUHK-NS-2200/Hong Kong/2019 (MN400355)	H	I	-	A	G	T	R	Q	G	E	D	K	G	D	L	Q	S	G
San Francisco	561/Gabon/2018 (MW506849)	Q	T	A	A	G	T	H	N	A	T	N	R	G	N	L	E	T	N
Sydney	RockvilleD1/United States/2012 (KY424328)	H	I	-	T	G	S	R	N	E	D	H	R	T	D	F	E	A	N
New Orleans	Virginia/Unites States/2010 (KX353958)	H	I	-	P	G	S	R	N	A	D	N	R	T	N	F	E	T	N
Apeldoorn	Iwate4/Japan/2008 (AB541274)	H	I	-	T	G	S	R	N	A	D	N	R	A	D	F	D	A	N
Osaka	Osaka/Japan/2007 (AB434770)	H	I	-	A	G	S	R	N	A	D	N	R	S	D	F	E	S	G
Farmington Hills	MD-2004/United States/2004 (DQ658413)	H	I	-	A	D	T	H	N	N	N	N	R	G	D	F	E	T	G
	Oxford/United Kingdom/2003 (AY588022)	H	I	-	A	G	T	H	N	N	N	N	R	G	D	F	E	T	G

Residues
Acidic
Basic
Polar neutral
Hydrophobic
Proline
Glycine
Cysteine

Variant	Strain (accession number)	Major antigenic sites involved in HBGA-blockade and neutralization																Subdominan		
		D					E					G						I		
		393	394	395	396	397	407	411	412	413	414	352	355	356	357	359	364	250	255	504
Hong Kong	CUHK-NS-2200/Hong Kong/2019 (MN400355)	E	N	P	H	F	S	R	T	G	H	S	A	A	D	A	N	Y	T	P
San Francisco	561/Gabon/2018 (MW506849)	D	T	A	H	R	S	R	N	T	P	Y	S	N	D	A	R	F	S	Q
Sydney	RockvilleD1/United States/2012 (KY424328)	S	T	T	H	R	S	R	N	T	H	Y	S	A	D	A	R	F	S	Q
New Orleans	Virginia/Unites States/2010 (KX353958)	S	A	T	P	R	S	R	N	I	H	Y	S	A	D	S	R	F	S	Q
Apeldoorn	Iwate4/Japan/2008 (AB541274)	D	T	A	H	R	S	R	N	S	H	Y	S	A	D	A	R	F	S	Q
Osaka	Osaka/Japan/2007 (AB434770)	S	T	T	H	R	N	R	T	G	H	L	S	A	D	A	R	Y	S	Q
Farmington Hills	MD-2004/United States/2004 (DQ658413)	N	G	T	H	Q	S	R	T	G	H	S	D	V	H	T	S	F	G	Q
	Oxford/United Kingdom/2003 (AY588022)	N	G	T	H	Q	S	R	T	G	H	S	D	V	H	T	S	F	G	Q

Variant	Strain (accession number)	Other sites with mutations specific to the new variants																			
		Shell	Protruding																		
		144	256	289	290	299	302	331	348	350	362	366	379	386	398	409	438	447	449	471	508
Hong Kong	CUHK-NS-2200/Hong Kong/2019 (MN400355)	V	A	D	L	F	H	Q	K	I	S	R	T	I	S	T	M	L	I	D	A
San Francisco	561/Gabon/2018 (MW506849)	I	T	S	V	Y	N	R	L	T	L	Q	Q	V	N	S	L	M	L	E	V
Sydney	RockvilleD1/United States/2012 (KY424328)	I	A	D	V	Y	N	Q	K	T	L	Q	Q	V	N	S	M	M	L	D	V
New Orleans	Virginia/Unites States/2010 (KX353958)	I	A	D	V	Y	N	Q	K	T	L	Q	Q	V	N	S	M	M	L	D	V
Apeldoorn	Iwate4/Japan/2008 (AB541274)	I	A	D	V	Y	N	Q	K	T	L	Q	Q	V	N	S	M	M	L	D	V
Osaka	Osaka/Japan/2007 (AB434770)	I	A	D	V	Y	N	Q	K	T	L	Q	Q	V	N	S	M	M	L	D	V
Farmington Hills	MD-2004/United States/2004 (DQ658413)	I	A	D	V	Y	N	Q	K	T	L	Q	Q	V	N	S	M	M	L	D	V
	Oxford/United Kingdom/2003 (AY588022)	I	A	D	V	Y	N	Q	K	T	L	Q	Q	V	N	S	M	M	L	D	V

Appendix Figure 3. Sequence alignment of major strain(s) from GII.4 variants used in immunoassays in this study. VLPs from the MD-2004/United States/2004 and RockvilleD1/United States/2012 viruses were used to generate mouse monoclonal antibodies (mAbs) (*3*). VLPs from Oxford/United Kingdom/2002, Osaka/Japan/2007, Iwate4/Japan/2008, Virginia/Unites States/2010, and RockvilleD1/United States/2012 were used to generate mouse hyperimmune sera (*14*). Because MD-2004/Unites States/2004 virus, which was used to produce mAbs for the Farmington Hills 2002 variant, has a mutation (G295D) on antigenic site A, sera raised against Oxford/United Kingdom/2003 was selected to test responses at the polyclonal level. Individual cells are colored based on the biochemical properties of the residues. HGBA, histo-blood group antigen; VLPs, virus-like particles.

Amino acid position	294			295			296			297			298			368			372			373				
**Nucleotide position**	**880**	**881**	**882**	**883**	**884**	**885**	**886**	**887**	**888**	**889**	**890**	**891**	**892**	**893**	**894**	**1102**	**1103**	**1104**	**1114**	**1115**	**1116**	**1117**	**1118**	**1119**		
San Francisco 2017	*A*			*G*			*S*			*H*			*N*			*E*			*N*			*N*			# Differences to San Francisco 2017	
	G	C	A	G	G	T	A	G	T	C	A	T	A	A	C	G	A	A	A	A	C	A	A	C	Nucleotide	Amino acid
Late Sydney 2012 (2018-2022)	***T***			*G*			*S*			*H*			*N*			*E*			*N*			***H***			4	2
	**A**	C	A	G	G	T	A	G	T	C	A	**C**	A	A	C	G	A	A	A	A	C	**C**	A	**T**		
Early Sydney 2012 (2012-2017)	***T***			*G*			*S*			***R***			*N*			*E*			***D***			***H***			5	4
	**A**	C	A	G	G	T	A	G	T	C	**G**	T	A	A	C	G	A	A	**G**	A	C	**C**	A	**T**		
Apeldoorn 2007	***T***			*G*			*S*			***R***			*N*			***A***			***D***			*N*			6	4
	**A**	C	A	G	G	T	A	G	T	C	**G**	T	A	A	C	G	**C**	**C**	**G**	A	C	A	A	**T**		
Farmington Hills 2002	*A*			*G*			***T***			*H*			*N*			***N***			*N*			*N*			5	2
	G	C	A	G	G	T	A	**C**	T	C	A	T	A	A	**T**	**A**	A	**C**	A	A	C	A	A	**T**		
Grimsby 1995	*A*			*G*			*S*			*H*			***D***			***T***			*N*			*N*			7	2
	G	C	A	G	G	**C**	A	G	T	C	A	T	**G**	A	**T**	**A**	**C**	**C**	A	A	C	A	A	**T**		

Appendix Figure 4. Comparison of consensus codon and amino acid sequences of antigenic site A from GII.4 variants. The amino acid sequences are shown in italic on the top and the corresponding codon (nucleotide) sequences are shown on the bottom. Mutations as compared to San Francisco 2017 consensus sequences are highlighted in bold and red.
